# DNA Barcoding and Analysis of Nutritional Properties as a Tool for Enhancing Traceability of Anchovies (*Engraulis encrasicolus* L.) Fished in the Italian Southern Adriatic Sea

**DOI:** 10.3390/genes16101219

**Published:** 2025-10-15

**Authors:** Maddalena de Virgilio, Domenico De Paola, Maria Selvaggi, Claudia Carbonara, Marco Ragni, Anna Caputi Jambrenghi, Francesco Giannico, Maria Antonietta Colonna, Simona Tarricone

**Affiliations:** 1 Institute of Bioscience and Bioresources, National Council of Research, Via Amendola 165/A, 70126 Bari, Italy; maddalena.devirgilio@cnr.it (M.d.V.); domenico.depaola@cnr.it (D.D.P.); 2 Department of Soil, Plant and Food Science, University of Bari Aldo Moro, Via Amendola 165/A, 70125 Bari, Italy; maria.selvaggi@uniba.it (M.S.); marco.ragni@uniba.it (M.R.); anna.caputijambrenghi@uniba.it (A.C.J.); simona.tarricone@uniba.it (S.T.); 3 Department of Medical and Surgical Sciences, University of Foggia, Via A. Gramsci 89/91, 71122 Foggia, Italy; 4 Department of Veterinary Medicine, University of Bari Aldo Moro, 70010 Valenzano, Italy; francesco.giannico@uniba.it

**Keywords:** *Engraulis encrasicolus*, anchovy, barcoding, traceability, flesh quality, chemical composition, fatty acid profile, nutritional value, seasonal variation, Adriatic Sea

## Abstract

**Background:** Anchovies (*Engraulis encrasicolus* L.) are a component of the Mediterranean diet and among the most fished species. Despite Italian consumers showing a strong preference and willingness to pay more for locally caught anchovies, cases of mislabeling with non-local or different species have been documented. Molecular techniques like DNA barcoding offer reliable species identification, even in processed products, where morphological traits are no longer detectable. This pilot study applied a DNA barcoding technique targeting the mitochondrial *cytochrome b* gene to authenticate anchovies caught in the Italian Southern Adriatic Sea. **Objectives:** The study evaluated seasonal variations in the chemical and nutritional composition of anchovies, particularly the fatty acid profiles, highlighting their health benefits. **Methods**: During 2021, two fish samplings of anchovies were conducted per season from two fishing areas in Southern Adriatic Sea, one sample was used for mitochondrial DNA analyses, the other was used for morphometric measurements, physical, bromatological and chemical analyses. **Results:** Fish collected in summer showed higher total weight and edible yield relative to those fished in winter (*p* < 0.05). Anchovies fished in summer contained the highest concentration of proteins (*p* < 0.05) as compared to those caught during winter and autumn, while, in turn, they showed the highest amount of fat (*p* < 0.01). Fillets from anchovies fished during spring and summer contained a greater (*p* < 0.05) concentration of polyunsaturated fatty acids, and n-3 fatty acids than samples collected in autumn and winter. **Conclusions:** This study paves the way for further investigation to refine and validate the genetic identification and nutritional features of anchovies caught in the Italian Southern Adriatic Sea and marketed to consumers.

## 1. Introduction

Fillets of pelagic marine species often show good nutritional value due to the high concentration of n-3 fatty acids (particularly eicosapentaenoic and docosahexaenoic acids), amino acids, and micronutrients necessary for human health [1]; therefore, their consumption in the human diet is highly recommended, especially for preventing heart diseases, dementia, mental decline, and many age-related illnesses [2,3,4]. Regarding heart disease, indices of atherogenicity (AI) and thrombogenicity (TI) are useful to predict the risk of atherosclerosis and platelet aggregation. Lower AI and TI rates implicate a greater protective potential for coronary artery disease; in fact, populations with a regular consumption of bluefishes are less vulnerable to heart disease [5].

The European anchovy (*Eugraulis encrasicolus*, Linnaeus 1758) is one of the most fished species in the Mediterranean Sea, since it represents almost 50% of the pelagic fishes caught, providing a significant economic boost to the fishing industry. This fish is commonly consumed in the Mediterranean diet, as it is considered a “poor man’s fish” due to its affordability and to the multiplicity of its traditional gastronomic uses, both fresh and processed [6,7,8].

Italian customers prefer anchovies fished along Italy’s coasts, as they perceive their freshness and high quality [9], willing to spend more in order to feel assured about the origin of the provenance that ensures the top quality of fish [10].

Due to consumers’ preference for local products, many cases of food fraud have occurred in the food supply chain. The local anchovy species, *Engraulis encrasicolus*, has often been replaced by *E. anchiota* [11] and, in some cases, a completely different species was (mis)labelled as anchovy [12]. Anchovies fished off the coasts of Africa, the Atlantic Ocean and the Aegean Sea were labelled as anchovies caught in Spanish waters [12]. Although it may not be visible to the public, the location of anchovy fishing affects the product’s nutritional profile [13], deceiving the consumer. For these reasons, it is essential to unambiguously identify commercially important fish species using biomolecular techniques.

As for anchovies, the small body size makes it difficult to identify visible morphological differences. Modern molecular biotechnologies serve as a valuable complement to traditional morphological methods of fish identification, helping to bridge gaps in taxonomic knowledge and solve the challenges associated with accurately assessing biodiversity. These molecular approaches enable the identification of cryptic species, clarification of species relationships, and proper taxonomic classification. As a result, a molecular approach such as DNA barcoding is essential for accurate species identification [14]. DNA barcoding offers a standardized and validated method for species-level identification on a global scale using genetic data. In this pilot study, DNA barcoding was applied to the mitochondrial *cytochrome b* (*Cyt-b*) gene commonly used as a molecular marker in marine fish species [15,16] to analyze nucleotide sequences and assess the geographic origin of anchovy samples collected from the fishing area of the Italian Southern Adriatic Sea, as well as their identification as *E. encrasicolus*. 

The objective of this study was to characterize European anchovies (*E. encrasicolus*) caught in the Italian Southern Adriatic Sea genetically, by DNA barcoding, and under the nutritional value of fillets, assessed for physical characteristics, chemical composition and fatty acid profiles.

## 2. Materials and Methods

### 2.1. Sample Collection

**Figure 1:**
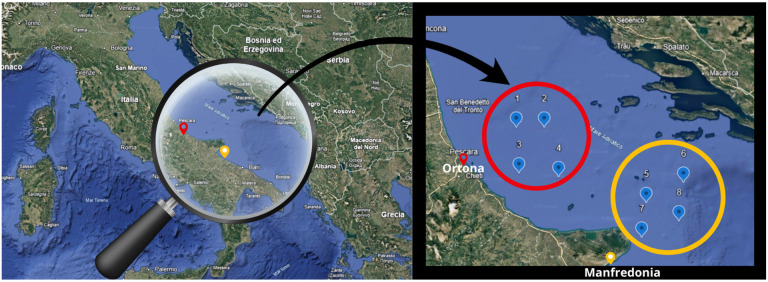
Investigated area (Ortona and Manfredonia) and sampling points: 1. 42°14′09″ N 14°53′46″ E; 2. 42°38′31″ N 14°41′25″ E; 3. 42°28′25″ N 14°32′06″ E; 4. 42°06′16″ N 14°56′48″ E; 5. 42°06′45″ N 16°19′43″ E; 6. 42°05′46″ N 16°45′38″ E; 7. 41°50′50″ N 16°19′17″ E; 8. 41°55′00″ N 16°47′35″ E.

Anchovies (*Engraulis encrasicolus* L.) were caught by traditional fishing methods in the Italian Southern Adriatic Sea (Figure 1); following capture, the fish were euthanized by immersion in ice-cold water (hypothermia), as required by the laws in force [17].

During 2021, two fish samplings were conducted per season, 45 days apart, from two fishing areas, as shown in Figure 1. Upon arrival to the port, the caught anchovies were transported to the laboratory under refrigerated conditions (0–4 °C) and used for morphometric measurements, physical, nutritional and chemical composition assessments, and the analysis of fatty acid composition.

### 2.2. DNA Extraction, Amplification and Sequencing

A total of 57 individuals was used for mitochondrial DNA (mt DNA) analyses, of which 24 were caught from sampling point 3 (Ortona area) and 33 from sampling point 7 (Manfredonia area), during fishing carried out in the winter season. DNA was extracted from the caudal fin using the Genomic DNA kit-Tissue Grisp (BIO-Cell Srl, Ciampino (RM), Italy), following the manufacturer’s recommendations. In brief, 100 mg of frozen caudal fins were homogenized using micropestles in Eppendorf tubes. The cells were lysed and proteins were denatured by adding chaotropic salts and proteinase K, and incubating the mixture at 60 °C for 20 min. After centrifugation at 14,000× *g* for 2 min, glass fibre spin columns were used to selectively bind the DNA from the homogenates. Contaminants, including proteins, divalent cations, unincorporated nucleotides, and enzyme inhibitors were thoroughly removed by washing the columns with a high-salt wash buffer containing ethanol, and with 30 s centrifugations at 14,000× *g*. Genomic DNA was eluted with 100 µL of water, followed by a final 30 s centrifugation at 14,000× *g*. For PCR, a fragment of 680 bp was amplified with an initial denaturation at 95 °C for 5 min, followed by 35 cycles (95 °C 60 s; 52 °C 60 s; 72 °C 60) with a final extension at 72 °C for 10 min, using the primers 5′-AACGACGCAGTAGTAGACC and 5′-GAGGAAGTATCACTCAGGC annealing to position 42 and 825 of the *Cyt-b* locus of *E. encrasicolus*, respectively [18]. PCR reactions were checked on a 1% agarose gel and purified with the PCR&gel Band Purification Kit Grisp (BIO-CELL Srl, Porto, Portugal). Amplicons were sequenced at the Eurofins Genomics Platform (Florence, Italy).

### 2.3. Data Treatment

DNA sequences were edited and trimmed at the 5′- and 3′- ends to remove low-quality DNA stretches. Multiple Sequence Alignment (MSA) was performed among 644 bp-fragments of the *Cyt-b* gene isolated in this study and *E. encrasicolus* sequences retrieved from GenBank, accession number KT246173 (from Morocco), FR851450 and FR851443 (from Italy), EU224051 and EU224052 (from France), EF427558 and EF427559 (from the Cantabrian Sea), EF 439527 and EF439526 (from the Western Mediterranean Sea). Sequences were aligned using the ClustalW algorithm [19], accessible via the KEGG web interface (https://www.genome.jp/tools-bin/clustalw (accessed on 28 September 2022)). MSA and the calculation of the number of haplotypes, polymorphic sites, singletons, and parsimony informative sites were carried out in DnaSP [20].

Two methods were used to generate phylogenetic reconstruction using the PhyML method available via the KEGG interface, including bootstrap support with 100 replicates: neighbor-joining and maximum likelihood [21]. The default substitution model in KEGG PhylML pipeline, HKY85, was used. The out-group used for this phylogenetic reconstruction was the *Cyt-b* gene of *Sardina pilchardus* (GenBank accession AF472582.1).

### 2.4. Biometric Measurements of Anchovies

For each catch, anchovy individuals were measured for the following parameters: weight of whole fish, of the fish without head and viscera, and of the edible portion, total length, fork length, head length, tail length and length to the first dorsal fin.

### 2.5. Colour and Textural Parameters in Anchovy Fillets

The colorimetric features (L* = lightness, a* = redness, and b* = yellowness) of the fish fillets were assessed using a Hunter Lab Miniscan™ XE Spectrophotometer (Model 4500/L, 45/0 LAV, 3.20 cm diameter aperture, 10° standard observer, focusing at 25 mm, illuminant D65/10; Hunter Associates Laboratory, Inc., Reston, VA, USA) by taking three readings for each sample along the whole fillet of the left side (in correspondence of the cranial, middle and caudal fin regions) [22]. The colour scale referred to was CIEL*a*b*, as described by Connoly et al. [23].

The rheological properties of the raw fish fillets were measured using an Instron 5544 Universal Testing Machine (Instron Corp., Canton, MA, USA). Texture Profile Analysis (TPA) was performed using a flat steel probe of 25 mm diameter, through a double compression test elaborated by the incorporated software. From the two fillets of each fish, three samples with a square surface (1 cm^2^) and a height of 1 cm were excised along the whole fillet. The mean values of three measurements of each test per fish were retained for statistical analysis [22].

### 2.6. Chemical and Fatty Acid Analysis of Fillets

As described by Tarricone et al. [24], on the day of analysis, the fillets were rapidly thawed, skinned, chopped, combined into a pool, homogenized, and lyophilized, after cooling to −80 °C for 48 h. AOAC procedures were used to assess the moisture, and ether extraction was used to determine the raw protein and ash content [25]. The total lipids were extracted according to the method described by Folch [26], using a 2:1 chloroform/methanol (*v*/*v*) solution to determine the fatty acid profile. The fatty acids were then methylated using a KOH/methanol 2N solution [27] and analyzed by gas chromatography (Shimadzu GC-17A, Kyoto, Japan) using a silicone-glass capillary column (70% cyanopropyl polysilphenylene-siloxane BPX 70 by Thermo Scientific, Waltham, MA, USA, length = 60 m, internal diameter = 0.25 mm, film thickness = 0.25 µm). The starting temperature was 135 °C for 7 min, then increased by 4 °C/min up to 210 °C, with a linear velocity near 37 cm/s. Fatty acids were identified by a comparison of retention times to authenticated standards (Food industry FAME mix, Restek Corporation, Bellefonte, PA, USA) for percentage area normalization. Fatty acids were expressed as a percentage (*wt*/*wt*) of total methylated fatty acids [22].

The lipid indices of fillets were determined by calculating the Atherogenic (AI) and Thrombogenic (TI) Indices as follows [28]:

AI = [(C12:0 + 4 × C14:0 + C16:0)] ÷ [ΣMUFA + Σn-6 + Σn-3];(1)

TI = [(C14:0 + C16:0 + C18:0)] ÷ [(0.5 × ΣMUFA + 0.5 × Σn-6 + 3 × Σn-3 + Σn-3)/Σn-6];(2)

where MUFA are monounsaturated fatty acids.

### 2.7. Statistical Analysis

Data were analyzed using the 9.1 2004 SAS software [29].

Each fish was considered as the experimental unit for the analysis of all data, while the two fish samplings within each season were considered as replicates. Data on the morphometric measurements and fillet quality traits (colour, TPA, chemical composition and fatty acid profile) were subjected to ANOVA and analyzed according to the following model:

y_ijk_ = μ+ α_i_ + β_j_ + (αβ)_ij_ + ε_ijk_ (3)

where μ represents the overall mean, α_i_ is the effect of fishing site (_i_: 1 to 2), β_j_ is the effect of seasons (_j_: 1 to 4), (αβ)_ij_ is the interaction between site and season, and ε_ijk_ accounts for random error.

The significance of differences among the groups was determined using Tukey’s test with *p* < 0.05 as the critical significance level. Since the interactions between site and season were not significant, the results are reported as least squares mean and standard errors of the mean (SEM) only for the seasons. When significant effects were found at *p* < 0.05, means were compared using Student’s *t*-test.

## 3. Results

### 3.1. DNA Barcoding

Multiple Sequence Alignment (MSA) performed among mtDNA *cytochrome b* sequences of *E. encrasicolus* accessions and the 57 sequences isolated in this study highlighted the presence of 51 polymorphic sites, and 24 singletons (Supplementary Figure S1).

In addition, the polymorphisms investigation defined 43 distinct *E. encrasiculus* mtDNA haplotypes, 27 of which were parsimoniously informative. High levels of genetic variation were found for haplotype diversity (h = 0.9779 ± 0.006) when compared to the haplotype diversity calculated in an analogous study [18].

Among the anchovies caught at the two fishing sites, we encountered high variability in positions 2, 3, and 4 of the MSA (see Supplementary Figure S1) corresponding to positions 140, 141, and 142 of the EF439527.1 GenBank accession where the following polymorphisms were found: AGG (11 individuals), TCA (24 individuals), ATG (3 individuals), ACA (8 individuals), TGG (4 individuals), AGC (1 individual), TCG (1 individual), AGA (2 individuals), AGT (1 individual), ACG (1 individual), and AAG (1 individual). All the sequences isolated in this study have been added to GenBank with accession numbers ranging from OK330182 to OK330235 (http://www.ncbi.nlm.nih.gov/GenBank/, accessed on 14 September 2025).

**Figure 2:**
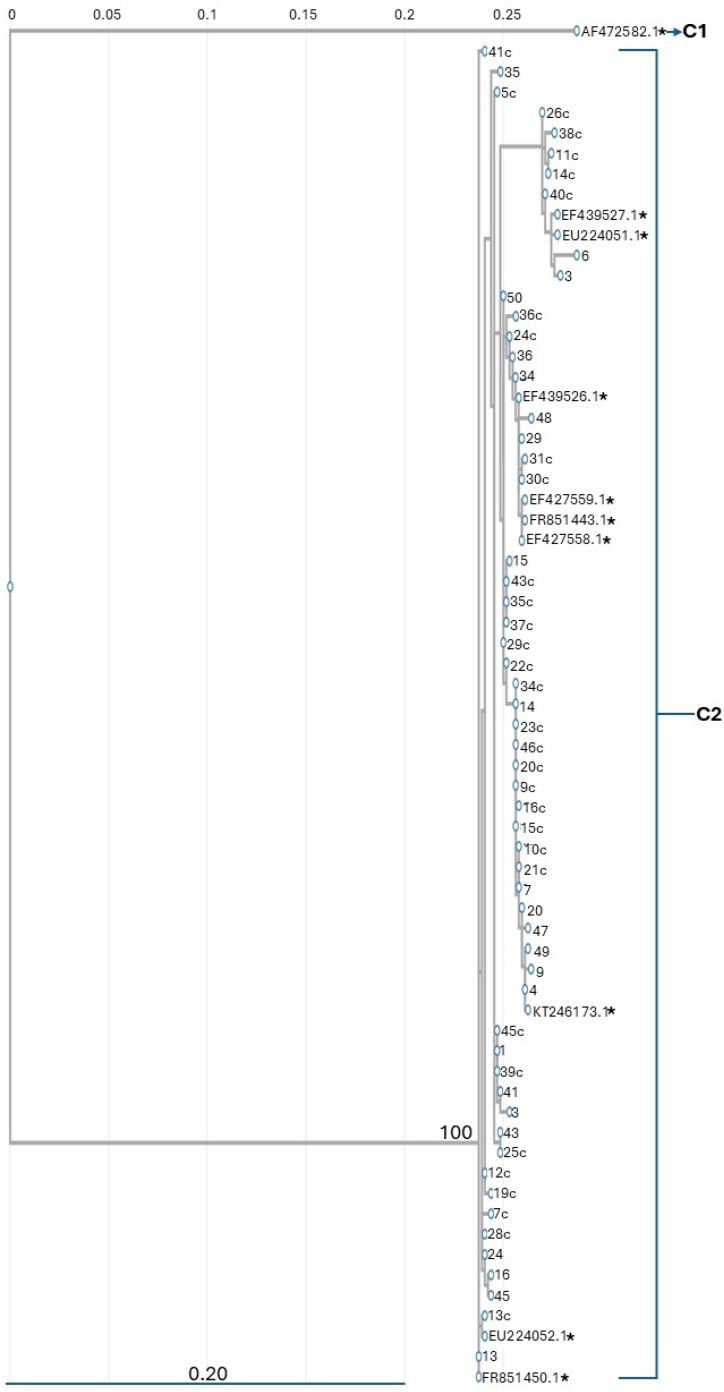
Maximum likelyhood-phylogenetic tree showing the relationship of *E. encrasicolus* mitochondrial DNA *Cytochrome b*. The number above the major node indicates bootstrap support for 100 replicates using PhyML. * indicates NCBI accessions of the *E. engranulis Cyt-b* sequences. **C1** and **C2** indicate Cluster 1 and Cluster 2, respectively.

A phylogenetic tree of 57 sequences isolated in this study and 9 NCBI accessions (KT246173, FR851450, FR851443, EU224051, EU224052, EF427558, EF427559, EF 439527, EF439526) was reconstructed from the amplification fragment included between positions 42 and 825 of the *Cyt-b* locus of *E. encrasicolus* using neighbour-joining and maximum likelihood methods. Both methods generated overlapping phylogenetic trees. The fragment included between positions 140 and 783 of the *Cyt-b* locus of *Sardina pilchardus* was used as the outgroup in the phylogenetic reconstruction. Two main clusters were formed with a bootstrap value of 100%. One cluster included only *S. pilchardus* sequences (Cluster 1, Figure 2), while the other cluster consisted of 57 sequences isolated in this study and 9 NCBI accessions of *E. encrasicolus* (Cluster 2, Figure 2). The genetic distance between clusters 1 and 2 was 0.2376. The genetic distance between the samples included in cluster 2 is ≤0.0015.

This finding indicates that the cluster of individuals coming from the Italian Southern Adriatic Sea overlapped the one detected for anchovies deriving from Morocco, Italy, France, the Cantabrian Sea, and the Western Mediterranean Sea previously deposited in GenBank.

### 3.2. Biometric Measurements of Anchovies

Table 1 shows the results of the biometric and morphological measurements carried out on anchovies caught during the different seasons. Fish collected in summer showed significantly higher total weight and edible yield relative to those fished in winter (*p* < 0.05).

### 3.3. Colour and Textural Parameters in Anchovy Fillets

Table 2 shows the results concerning the colour features and the texture parameter assessment (TPA) of anchovies caught during the different seasons. There were no significant effects of season on the colour indices or TPA values of anchovy fillets.

### 3.4. Chemical and Fatty Acid Analysis of Fillets

The chemical composition of anchovy fillets caught during the different seasons is shown in Table 3. Anchovies fished in summer contained a higher concentration of proteins (*p* < 0.05) than those caught during winter and autumn, while, in turn, they showed the highest amount of fat (*p* < 0.01).

The seasonal variation in the fatty acid composition of anchovy fillets is shown in Table 4. Fish collected in autumn and winter contained a significantly (*p* < 0.05) higher concentration of palmitic acid (C16:0) than anchovies caught in summer. The same trend was observed for the amount of total saturated fatty acids (SFAs), which was higher (*p* < 0.01) in autumn and winter than in summer.

With regard to the monounsaturated fatty acids (MUFAs), statistical differences were observed only for the concentration of tetradecenoic acid (C14:1), which was significantly greater in anchovy fillets caught in autumn (*p* < 0.05) than in winter and spring.

Fillets from anchovies fished during spring and summer contained a greater (*p* < 0.05) concentration of eicosadienoic (C20:2 n-6), eicosatrienoic (C20:3 n-6), docosahexaenoic (C22:6 n-3), total polyunsaturated fatty acids (PUFAs), and total n-3 fatty acids than the samples collected in autumn and winter.

The Atherogenic and Thrombogenic indices were unaffected by the fishing season.

## 4. Discussion

DNA barcoding has proven to be a valuable tool in determining the geographic provenience of anchovies (family *Engraulidae*), especially in the context of food traceability, conservation, and fisheries management [16]. This pilot investigation was performed on anchovies fished in the Italian Southern Adriatic Sea. The phylogenetic reconstructions based on a 680 bp fragment of the *Cyt-b* locus allowed the identification of all the individuals examined as *E. encrasicolus*. A genetic distance greater than 3% indicates that each clade of a phylogenetic tree is a distinct species [30], and in this study, we detected a genetic distance of 0.237 among *S. pilchardus* and *E. encrasicolus Cyt-b* gene sequences. At the same time, the genetic distance between *E. encrasicolus Cyt-b* accessions in GenBank and the sequences isolated in this study was ≤0.0015.

Notably, exclusively within the *Cyt-b* sequence of the individuals caught at both fishing sites, high variability was detected in *Cyt-b* positions 19, 20, and 21, where the following polymorphisms were found: AGG, TCA, ATG, ACA, TGG, AGC, TCG, AGA, AGT, ACG, and AAG. These polymorphisms were absent in the NCBI accessions used as the reference clade; therefore, they may be considered as identifiers of the provenance of *E. encrasicolus* from the Italian Southern Adriatic Sea. Even if indicative, the findings of this pilot study do not provide conclusive evidence that these polymorphisms can unambiguously identify the provenance of European anchovies from the Southern Adriatic Sea. Further population genetic studies on Mediterranean stocks of anchovies are needed to demonstrate that the *cytochrome b* gene polymorphisms identified herein can be considered as indicators of the origin of *E. encrasicolus* anchovies from the Italian Southern Adriatic Sea.

The physical and chemical characteristics of Adriatic European fish fillets change throughout the year, depending on several factors, such as the life stage of the fish, the season, water temperature and salinity, and feed resources available [31,32,33]. In the Adriatic Sea, during their spawning season, from April to September [34], anchovies increase their feed intake to meet a higher energy demand. The high availability of decapod crustacean larvae together with the presence of a great quantity of fish eggs during these months guarantees the higher energy needed for reproduction. Similar results were noted for this species on the Algerian coast [35] and in Izmir Bay [36], where during the spawning period, small copepods were gradually replaced by larger prey. This kind of feeding may have supported a significant increase in fish weight during summer.

An increase in the lipid concentration in fillets was observed during winter, in correspondence with the lowest water temperatures. Ruyter et al. [37] reported that changes in water temperature may affect the lipid composition of fish cell membranes due to a process known as homoviscous adaptation. As a consequence, low water temperatures lead to a greater membrane rigidity that may be contrasted by the deposition of higher amounts of unsaturated fatty acids, which increase membrane fluidity. Özyurt et al. [38] recorded decreased levels of unsaturated fatty acids in sea bream (*Sparus aurata*) caught during the winter season, attributing this decrease to the catabolism of saturated fat to satisfy the increased metabolic energy needs during the cold season. In contrast, in this study, the muscle tissue of anchovy fillets caught during autumn and winter showed the highest concentrations of SFA. This may be explained by the higher average water temperature of the Southern Adriatic Sea as compared to other seas, that is, around 14–17 °C in January, the coldest month of the year. The amount of total fat, the distribution among the lipid classes and the fatty acid profile in fillets vary widely within and among fish species, depending on several factors, such as food availability, season, location, sex, and age, which has been well-documented by many authors [39,40,41,42]. The most abundant saturated fatty acid found in the edible portion of anchovy fillets was palmitic acid (C16:0), a primary SFA in this species, which ranged from 29.55% to 32.08%, in accordance with previous findings [43,44]. In the present study, the fatty acid profile of European anchovy fillets was similar to that of anchovies fished in various seas [42,45,46,47]. In comparison with our results, higher levels of C16:0 were observed for anchovies caught in the Marmara Sea (range 22.27–35.43%) [44] as well as in the Mediterranean Sea (17.86–35.63%) [48].

Docosahexaenoic acid (C22:6 n-3; DHA) was the most abundant PUFA in the fillets, followed by eicosapentaenoic acid (C20:5 n-3; EPA), as found for anchovies captured in the Black and Mediterranean Seas. In accordance with our results, other studies observed a variation in the content of docosahexaenoic acid in relation to the month of capture [47,49]. Tufan et al. [45] reported the highest amounts of DHA in fish caught during February and April, while the lowest values were found in September and October. The highest level of DHA was recorded in summer, while the lowest was in winter, in accordance with the results of other studies [43]. DHA is a fatty acid important for human health, since it has well-documented effects on the prevention of coronary and arterial diseases [50,51]. In this study, the total PUFA concentration in anchovies ranged from 33.0% to 36.9% in the groups; this amount is approximately equal to 1.8 g/100 g of an edible portion of fresh anchovies, which is greater than the recommended daily intake of 0.4 g of total PUFAs, as reported by other authors [52,53].

The n-3/n-6 ratio is considered a useful indicator for comparing the relative nutritional values of fish oils [54]. In this study, we found a high n-3/n-6 ratio in anchovies that remained quite constant during the year, showing to be unaffected by seasonal variations. The nutritional significance of the n-6⁄n-3 ratio has been underlined by several authors [51,54] as one of the key elements of a healthy diet. The n-6/n-3 ratio was low in all seasons, showing values ranging between 0.08 and 0.09. The balance between the n-6/n-3 PUFA ratio is important in improving the inflammatory response, and maintaining a 1:1 ratio of n-6/n-3 PUFAs has been reported to inhibit the production of inflammatory factors such as TNF-α and IL-6, which can reduce inflammation and contribute to a healthy intestinal microenvironment [55]. The PUFA/SFA ratio recommended for human health is 0.45 [54,55]; in this study, the PUFA/SFA ratio ranged from 0.66 to 0.83 throughout the seasons, which is higher than the recommended value. This observation suggests potential beneficial effects of anchovy consumption in the human diet. Lloret et al. [54] reported that the dietary consumption of several Mediterranean pelagic species, including anchovy, sardine, bluefin tuna, and Atlantic mackerel, is very important for human health due to the high amount of n-3 fatty acids that, in turn, depends on the characteristics of the habitats, including the quantity and quality of prey, which consists of plankton for small pelagic species, and benthic fauna for many benthic species. This may be explained by the fact that n-3 fatty acids are produced by phytoplankton and marine plants (algae and seagrass meadows), eaten by primary consumers in benthic habitats that are included through the food chain.

The indices of atherogenicity and thrombogenicity did not differ among the seasons. In the current study, AI rates ranged from 0.76 to 0.93, which is in accordance with similar results reported in other studies for marine fish species [5,56,57]. The TI values fell within the range of 0.31–0.38, which is very low, as compared to previous findings [58,59], indicating that the consumption of anchovies is beneficial to human health [60].

## 5. Conclusions

This study contributes to generating knowledge of DNA barcoding as a valuable tool in determining the geographic provenience of anchovies (*E. encrasicolus*), to guarantee food traceability for consumer protection. The *Cyt b* sequence of the anchovies caught in the two fishing sites showed high variability in positions 19, 20 and 21, evidencing polymorphisms absent in the NCBI accessions used as the reference clade.

Seasonal variations in the chemical and fatty acid profiles of anchovies did not affect the overall nutritional quality of this fish species, whose consumption is highly recommended for the health benefits.

The information generated in this study paves the way for further investigations to refine and validate, in independent larger datasets, the genetic and nutritional features of anchovies caught in the Italian Adriatic Sea.

## Figures and Tables

**Table 1:** Biometric and morphological measurements of anchovies caught in different seasons.

Parameters	Winter	Spring	Summer	Autumn	SEM ^1^	*p*-Value
Total body weight (g)	14.11 ^b^	15.04 ^ab^	16.93 ^a^	15.70 ^ab^	1.264	0.034
Edible yield (%)	8.48 ^b^	9.23 ^ab^	9.92 ^a^	9.34 ^ab^	0.137	0.039
Total length (cm)	12.70	12.90	12.87	12.34	0.211	0.108
Fork length (cm)	12.00	11.30	11.97	11.41	0.032	0.097
Head length (cm)	1.90	2.10	1.94	2.24	0.428	0.135
Tail length (cm)	1.70	1.50	1.90	1.48	0.312	0.261
Length at the first dorsal fin (cm)	5.40	5.30	5.33	5.08	0.014	0.139

^1^ SEM: Standard error of means; ^a, b^: *p* < 0.05.

**Table 2:** Colour features and TPA in fillets from anchovies caught in different seasons.

Parameters	Winter	Spring	Summer	Autumn	SEM ^1^	*p*-Value
L* (lightness)	34.15	37.93	35.34	37.77	1.291	0.184
a* (redness)	1.53	1.27	1.18	1.45	0.042	0.127
b* (yellowness)	4.57	4.79	5.45	5.40	0.436	0.049
Maximum compression 2nd cycle (mm)	5.85	6.01	5.84	5.99	0.090	0.121
Springiness (mm)	3.91	4.59	3.96	4.68	0.406	0.099
Cohesion force resilience (N/m)	0.60	0.75	0.72	0.77	0.076	0.084
Chewiness (N*mm)	6.53	7.73	6.89	7.09	0.503	0.107

^1^ SEM: Standard error of means.

**Table 3:** Chemical composition of fillets of anchovies caught in different seasons (%).

Parameters	Winter	Spring	Summer	Autumn	SEM ^1^	*p*-Value
Moisture	72.45	72.95	72.70	72.41	0.250	0.238
Crude Protein	18.48 ^b^	18.77 ^ab^	19.59 ^a^	18.37 ^b^	0.051	0.037
Lipid	4.89 ^A^	3.65 ^B^	3.69 ^B^	5.24 ^A^	0.018	0.007
Ash	2.09	2.09	2.18	1.56	0.283	0.059
N Free-Extract	2.09	2.54	1.84	2.42	0.318	0.055

^1^ SEM: Standard error of means; ^a, b^: *p* < 0.05; ^A, B^: *p* < 0.01.

**Table 4:** Fatty acid profile (% total FA methyl esters) of fillets in anchovies caught in different seasons.

Fatty Acids	Winter	Spring	Summer	Autumn	SEM ^1^	*p*-Value
C12:0	0.07	0.08	0.05	0.04	0.018	0.078
C13:0	0.03	0.03	0.04	0.08	0.024	0.081
C14:0	3.08	3.19	2.82	3.75	0.392	0.127
C15:0	0.84	0.89	0.63	0.59	0.150	0.116
C16:0	32.06 ^a^	30.89 ^ab^	29.55 ^b^	32.08 ^a^	0.200	0.043
C17:0	1.09	1.16	0.84	1.01	0.138	0.177
C18:0	7.09	6.95	5.91	5.86	0.658	0.088
C20:0	0.06	0.05	0.07	0.07	0.010	0.065
C21:0	0.23	0.33	0.35	0.38	0.065	0.112
C22:0	0.04	0.04	0.04	0.04	0.001	0.073
C23:0	5.67	4.18	4.25	5.19	0.729	0.135
SFAs	50.26 ^A^	47.79 ^AB^	44.55 ^B^	49.09 ^A^	1.464	0.008
C14:1	0.12 ^b^	0.16 ^b^	0.28 ^ab^	0.52 ^a^	0.180	0.034
C15:1	0.09	0.11	0.07	0.28	0.096	0.097
C16:1 *trans*	0.09	0.12	0.11	0.14	0.021	0.114
C16:1 *cis*	1.37	1.34	1.54	1.18	0.148	0.125
C17:1	0.34	0.35	0.34	0.38	0.019	0.087
C18:1 n-9 *trans*	0.18	0.14	0.14	0.15	0.051	0.144
C18:1 n-9 *cis*	12.63	13.32	12.25	12.11	0.542	0.078
C20:1 n-9	0.19	0.19	0.15	0.14	0.026	0.201
C22:1 n-9	0.86	0.61	0.92	0.63	0.158	0.099
C24:1 n-9	0.74	0.52	0.74	0.94	0.172	0.067
MUFAs	16.61	16.86	16.54	16.57	0.146	0.107
C18:2 n-6 *trans*	0.13	0.09	0.11	0.12	0.017	0.081
C18:2 n-6 *cis*	1.25	1.18	1.13	1.31	0.079	0.096
C18:3 n-6	0.26	0.25	0.24	0.24	0.010	0.077
C18:3 n-3	0.58	0.71	0.57	0.59	0.066	0.149
C20:2 n-6	0.22 ^b^	0.39 ^a^	0.41 ^a^	0.18 ^b^	0.114	0.042
C20:3 n-6	0.45 ^b^	0.63 ^a^	0.62 ^a^	0.45 ^b^	0.077	0.037
C20:4 n-6	0.11	0.14	0.09	0.11	0.021	0.102
C20:3 n-3	0.07	0.07	0.06	0.06	0.006	0.084
C22:2 n-6	0.27	0.23	0.15	0.13	0.166	0.079
C20:5 n-3	10.31	10.24	10.33	10.32	0.039	1.021
C22:5 n-3	3.12	3.19	3.41	3.41	0.147	1.104
C22:6 n-3	16.04 ^b^	19.15 ^a^	19.92 ^a^	17.09 ^b^	0.660	0.047
∑ PUFA	33.01 ^b^	36.27 ^a^	36.92 ^a^	34.08 ^b^	1.662	0.041
n-6	2.69	2.91	2.73	2.54	0.153	0.064
n-3	30.12 ^b^	33.36 ^a^	34.29 ^a^	31.44 ^b^	1.765	0.032
n-6/n-3	0.09	0.09	0.08	0.08	0.009	0.087
n-3/n-6	11.20 ^b^	11.46 ^ab^	12.56 ^a^	12.37 ^ab^	1.185	0.041
PUFA/SFA	0.66	0.70	0.83	0.64	0.046	0.074
AI	0.90	0.86	0.76	0.93	0.071	0.075
TI	0.38	0.36	0.31	0.36	0.033	0.098

^1^ SEM: Standard error of means; SFAs—saturated fatty acids (sum of C12:0 + C13:0 + C14:0 + C15:0 + C16:0 + C17:0 + C18:0 + C20:0 + C21:0 + C22:0 + C23:0); MUFAs—monounsaturated fatty acids (sum of C14:1 + C15:1 + C16:1 trans + C16:1 cis + C17:1 + C18:1 n-9 cis + C18:1 n-9 trans + C20:1 n-9 + C22:1 n-9 + C24:1 n-9); PUFAs—polyunsaturated fatty acids (sum of n-6 + n-3); n-6 = C18:2 n-6 cis + C18:2 n-6 trans + C18:3 n-6 + C20:2 n-6 + C20:3 n-6 + C20:4 n-6 + C22:2 n-6; n-3 = C18:3 n-3 + C20:3 n-3 + C20:5 n-3 + C22:5 n-3 + C22:6 n-3; AI—Atherogenic index; TI—Thrombogenic index; ^a, b^: *p* < 0.05; ^A, B^: *p* < 0.01.

## Data Availability

All the sequences isolated in this study were stored in GenBank with accession numbers ranging from OK330182 to OK330235.

## Supplementary Materials

The following supporting information can be downloaded at https://www.mdpi.com/article/10.3390/genes16101219/s1, Figure S1: MSA between mtDNA *Cyt-b E. encrasicolus* accessions available at GenBank (KT246173, KT246289 FR851450, FR851450, FR851443 EU224051, EU224052 EF427558, EF427559 EF 439527, EF439526) and the 57 sequences isolated in this study.
