# Supplementary figures and images for: DNA Barcoding and Analysis of Nutritional Properties as a Tool for Enhancing Traceability of Anchovies (Engraulis encrasicolus L.) Fished in the Italian Southern Adriatic Sea
Source: Genes (Basel). 2025 Oct 15;16(10):1219. doi: 10.3390/genes16101219 (PMC12563354; doi:10.3390/genes16101219)

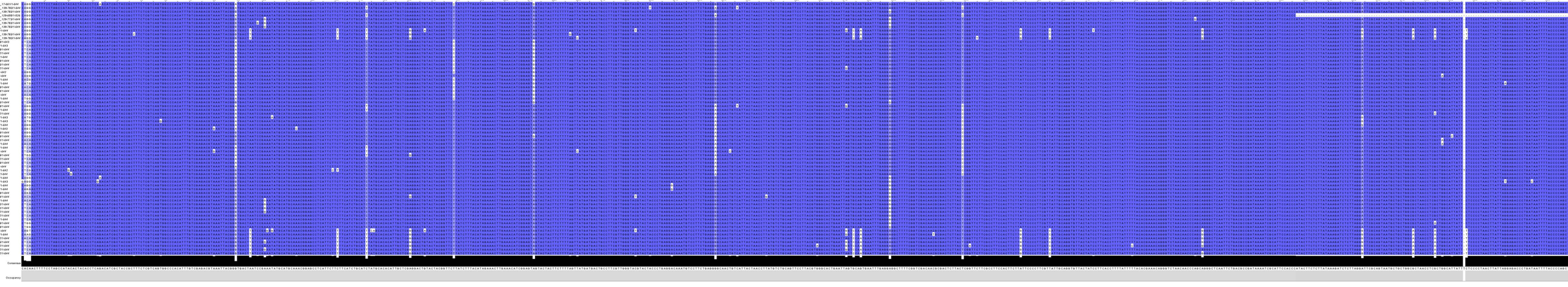

Supplement: Supplementary file 1 [file genes-16-01219-s001.zip › genes-3887347-supplementary.pdf]
